# 2-(6-Hy­droxy-1-benzo­furan-3-yl)acetamide

**DOI:** 10.1107/S1600536813033436

**Published:** 2013-12-24

**Authors:** D. B. Arunakumar, G. Krishnaswamy, S. Sreenivasa, K. J. Pampa, N. K. Lokanath, P. A. Suchetan

**Affiliations:** a Department of Studies and Research in Chemistry, Tumkur University, Tumkur, Karnataka 572 103, India; b Department of Studies in Physics, University of Mysore, Manasagangotri, Mysore, India; c Department of Studies and Research in Chemistry,U.C.S, Tumkur University, Tumkur, Karnataka 572 103, India

## Abstract

In the title compound, C_10_H_9_NO_3_, the dihedral angle between the benzo­furan ring system (r.m.s. deviation for the non-H atoms = 0.009 Å) and the –C—C(O)—N– segment is 83.76 (1)°. In the crystal, mol­ecules are linked by N—H⋯O and O—H⋯O hydrogen bonds, generating (001) sheets, which feature *C*(4) and *C*(10) chains.

## Related literature   

For a related structure and background to benzo­furans, see: Arunakumar *et al.* (2014 ▶).
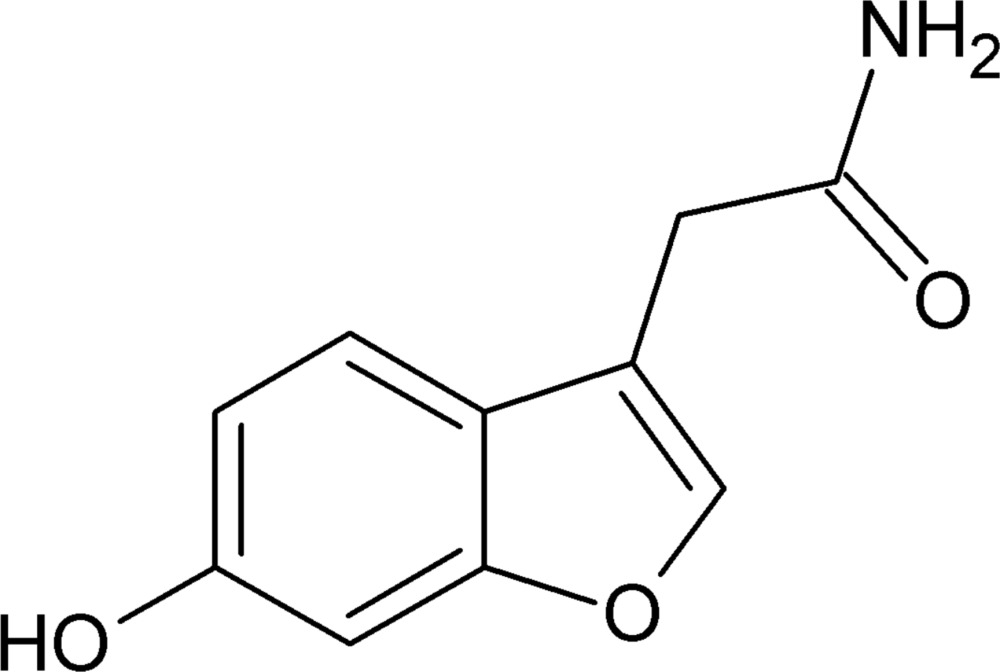


## Experimental   

### 

#### Crystal data  

C_10_H_9_NO_3_
*M* *_r_* = 191.18
Orthorhombic, 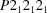

*a* = 5.0939 (3) Å
*b* = 9.3629 (5) Å
*c* = 18.7422 (10) Å
*V* = 893.88 (9) Å^3^
*Z* = 4
Cu *K*α radiation
μ = 0.89 mm^−1^
*T* = 293 K
0.36 × 0.29 × 0.24 mm

#### Data collection  

Bruker APEXII CCD diffractometer
Absorption correction: multi-scan (*SADABS*; Bruker, 2009 ▶) *T*_min_ = 0.770, *T*_max_ = 0.808
8714 measured reflections
1459 independent reflections
1446 reflections with *I* > 2σ(*I*)
*R*_int_ = 0.036

#### Refinement  

*R*[*F* ^2^ > 2σ(*F* ^2^)] = 0.033
*wR*(*F* ^2^) = 0.081
*S* = 1.14
1459 reflections
136 parameters
H atoms treated by a mixture of independent and constrained refinement
Δρ_max_ = 0.15 e Å^−3^
Δρ_min_ = −0.28 e Å^−3^
Absolute structure: Flack (1983 ▶), 1927 Friedel pairs
Absolute structure parameter: −0.2 (2)

### 

Data collection: *APEX2* (Bruker, 2009 ▶); cell refinement: *SAINT-Plus* (Bruker, 2009 ▶); data reduction: *SAINT-Plus* and *XPREP* (Bruker, 2009 ▶); program(s) used to solve structure: *SHELXS97* (Sheldrick, 2008 ▶); program(s) used to refine structure: *SHELXL97* (Sheldrick, 2008 ▶); molecular graphics: *Mercury* (Macrae *et al.*, 2008 ▶); software used to prepare material for publication: *SHELXL97*.

## Supplementary Material

Crystal structure: contains datablock(s) I, New_Global_Publ_Block. DOI: 10.1107/S1600536813033436/hb7172sup1.cif

Structure factors: contains datablock(s) I. DOI: 10.1107/S1600536813033436/hb7172Isup2.hkl

Additional supporting information:  crystallographic information; 3D view; checkCIF report

## Figures and Tables

**Table 1:** Hydrogen-bond geometry (Å, °)

*D*—H⋯*A*	*D*—H	H⋯*A*	*D*⋯*A*	*D*—H⋯*A*
O3—H*O*3⋯O1^i^	0.82	1.92	2.7006 (16)	158
N1—H1*N*⋯O3^ii^	0.92 (3)	2.08 (3)	2.969 (2)	162 (2)
N1—H2*N*⋯O1^iii^	0.92 (3)	2.03 (3)	2.8958 (18)	156 (2)

Symmetry codes: (i) 
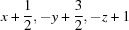
; (ii) 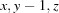; (iii) 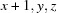.

**Fig. 1.**
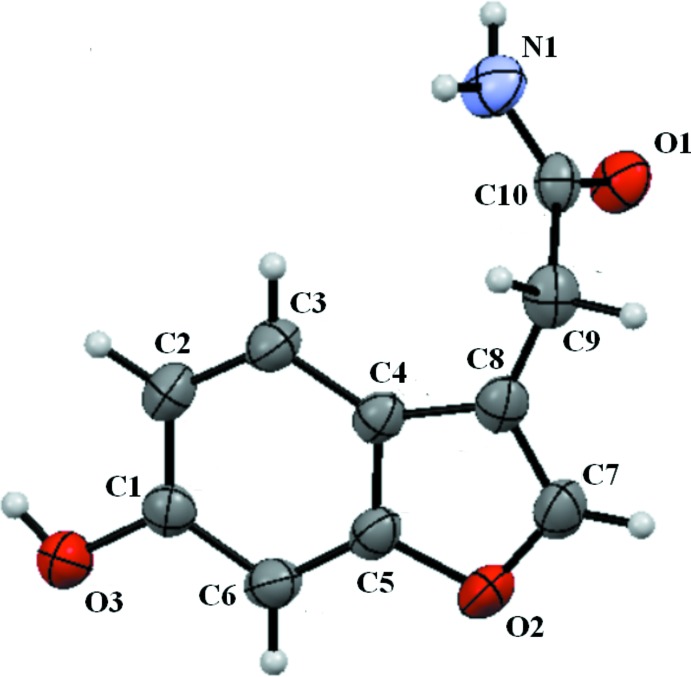
Molecular structure of the title compound, showing displacement ellipsoids drawn at the 50% probability level.

**Fig. 2.**
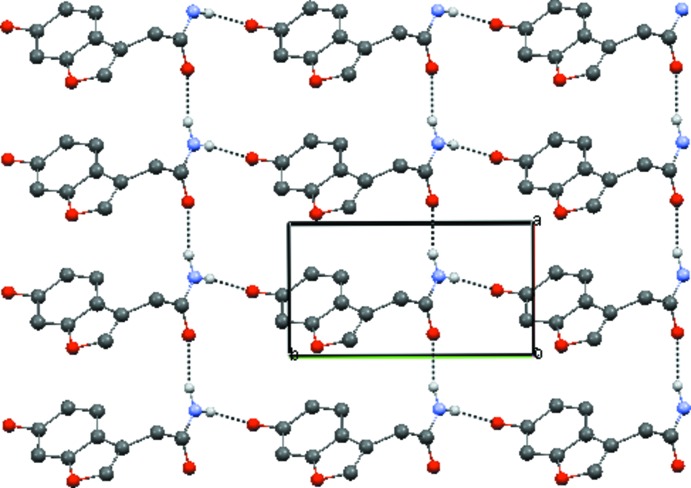
Linking of molecules in the crystal structure through N—H···O hydrogen bonds into C(4) and C(10) chains. H-atoms not involved in H-bonding are omitted for clarity.

**Fig. 3.**
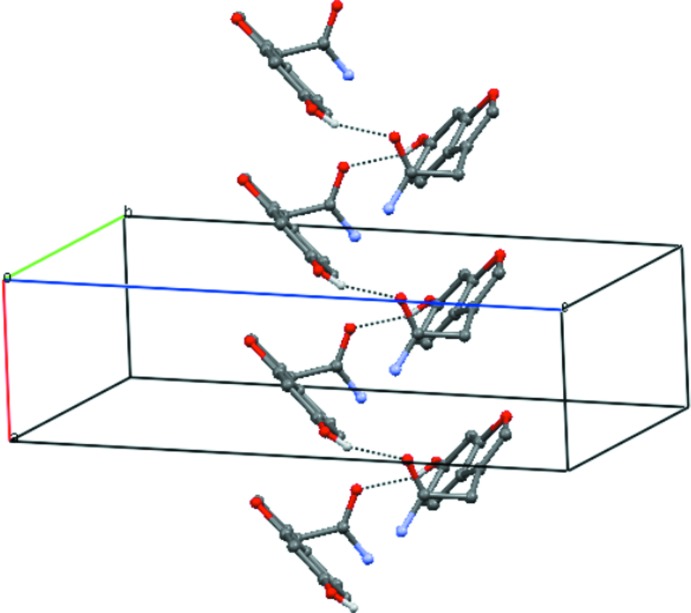
Formation of helical structure through O—H···O hydrogen bonds.

**Fig. 4.**
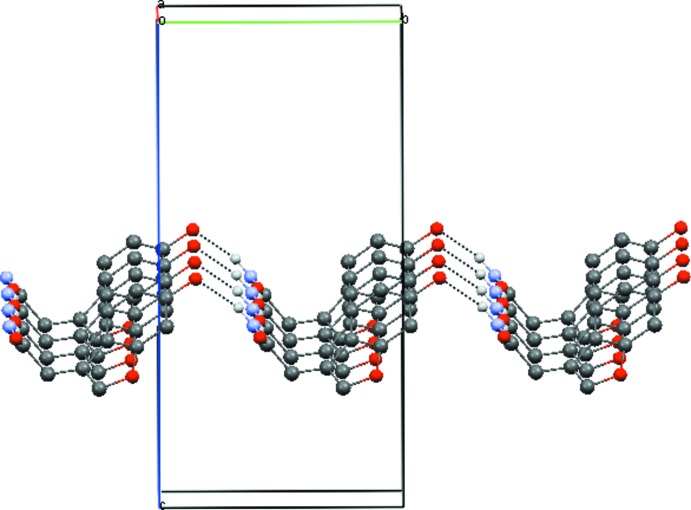
Zig-zag pattern observed in the crystal structure.

## supplementary crystallographic information

### 1. Comment

As part of our ongoing structural studies of functionalsied benzofuran ring systems at the C-2 position (Arunakumar *et al.*, 2014), we now describe the structure of the title compound, (I).

In the title compound, C_10_H_9_NO_3_, the benzofuran ring is almost planar ((r.m.s. deviation for the non-H atoms = 0.009 Å) (Fig. 1). The dihedral angle between the the two planes defined by the benzofuran ring and the –C—C(O)—N– segment is 96.24 (1)°. Compared to this, the dihedral angle between the benzofuran ring and the –C—C—N—O segment in the related structure (Arunakumar *et al.*, 2014) is 2.85 (1)°. In the crystal structure, the molecules are linked to one another through strong N1—H1N···O3 and N1—H2N···O1 hydrogen bonds generating C(4) and C(10) chains (Fig. 2). The molecules are further connected into C(10) chains through strong O3—HO3···O1 hydrogen bonds forming helical structure (Fig. 3). Linking of molecules in zig zag pattern through N1—H1N···O3 H-bonds is shown in Fig. 4.

### 2. Experimental

(6-Hydroxy-1-benzofuran-3-yl)acetic acid (0.015 mmol) was taken in a round bottom flask containing *N*,*N*-dimethyl formamide (15 ml)·To this 1, 1-carbonydiimadazole (0.023 mmol) was added at 273 K. The reaction mixture was stirred for 45 minutes. Ammonium acetate (0.046 mmol) and triethyl amine (1 ml) were added, the reaction mixture was stirred at room temperature overnight. The completion of the reaction was monitored by thin layer chromatography. After completion of the reaction, the reaction mixture was diluted with ethylacetate and the organic layer was washed with water followed by brine solution. The organic layer was dried over anhydrous sodium sulfate and concentrated to give the crude product. It was further purified by column chromatography by using Ethyl acetate and petroleum ether (8:2) as eluent.

Colourless prisms were obtained from the solvent system: ethyl acetate / methanol (4:1) at room temperature.

### 3. Refinement

The H atoms were positioned with idealized geometry using a riding model with C—H = 0.93–0.97 Å and O—H = 0.82 Å. The H atoms of the NH_2_ group were located in a difference map and later refined freely. The isotropic displacement parameters for all H atoms were set to 1.2 times *U*_eq_ (Carbon) and 1.5 times *U*_eq_ (Oxygen)..

### Crystal data

**Table:**

C_10_H_9_NO_3_	Prism
*M**_r_* = 191.18	*D*_x_ = 1.421 Mg m^−^^3^
Orthorhombic, *P*2_1_2_1_2_1_	Melting point: 533 K
Hall symbol: P 2ac 2ab	Cu *K*α radiation, λ = 1.54178 Å
*a* = 5.0939 (3) Å	Cell parameters from 1234 reflections
*b* = 9.3629 (5) Å	θ = 4.7–64.3°
*c* = 18.7422 (10) Å	µ = 0.89 mm^−^^1^
*V* = 893.88 (9) Å^3^	*T* = 293 K
*Z* = 4	Prism, colourless
*F*(000) = 400	0.36 × 0.29 × 0.24 mm

### Data collection

**Table:**

Bruker APEXII CCD diffractometer	1459 independent reflections
Radiation source: fine-focus sealed tube	1446 reflections with *I* > 2σ(*I*)
Graphite monochromator	*R*_int_ = 0.036
φ and ω scans	θ_max_ = 64.3°, θ_min_ = 4.7°
Absorption correction: multi-scan (*SADABS*; Bruker, 2009)	*h* = −5→5
*T*_min_ = 0.770, *T*_max_ = 0.808	*k* = −10→10
8714 measured reflections	*l* = −21→21

### Refinement

**Table:**

Refinement on *F*^2^	Secondary atom site location: difference Fourier map
Least-squares matrix: full	Hydrogen site location: inferred from neighbouring sites
*R*[*F*^2^ > 2σ(*F*^2^)] = 0.033	H atoms treated by a mixture of independent and constrained refinement
*wR*(*F*^2^) = 0.081	*w* = 1/[σ^2^(*F*_o_^2^) + (0.0488*P*)^2^ + 0.1043*P*] where *P* = (*F*_o_^2^ + 2*F*_c_^2^)/3
*S* = 1.14	(Δ/σ)_max_ < 0.001
1459 reflections	Δρ_max_ = 0.15 e Å^−^^3^
136 parameters	Δρ_min_ = −0.28 e Å^−^^3^
0 restraints	Absolute structure: Flack (1983), 1927 Friedel pairs
Primary atom site location: structure-invariant direct methods	Absolute structure parameter: −0.2 (2)

### Special details

**Table:**

Geometry. All s.u.'s (except the s.u. in the dihedral angle between two l.s. planes) are estimated using the full covariance matrix. The cell s.u.'s are taken into account individually in the estimation of s.u.'s in distances, angles and torsion angles; correlations between s.u.'s in cell parameters are only used when they are defined by crystal symmetry. An approximate (isotropic) treatment of cell s.u.'s is used for estimating s.u.'s involving l.s. planes.
Refinement. Refinement of *F*^2^ against ALL reflections. The weighted *R*-factor *wR* and goodness of fit *S* are based on *F*^2^, conventional *R*-factors *R* are based on *F*, with *F* set to zero for negative *F*^2^. The threshold expression of *F*^2^ > 2σ(*F*^2^) is used only for calculating *R*-factors(gt) *etc*. and is not relevant to the choice of reflections for refinement. *R*-factors based on *F*^2^ are statistically about twice as large as those based on *F*, and *R*- factors based on ALL data will be even larger.

### Fractional atomic coordinates and isotropic or equivalent isotropic displacement parameters (Å^2^)

**Table:**

	*x*	*y*	*z*	*U*_iso_*/*U*_eq_	
H2N	0.766 (5)	0.410 (3)	0.6264 (14)	0.072 (7)*	
H1N	0.587 (5)	0.324 (3)	0.5742 (14)	0.069 (7)*	
C1	0.4600 (3)	1.02627 (16)	0.55047 (8)	0.0361 (4)	
C2	0.6218 (3)	0.90706 (18)	0.54019 (9)	0.0404 (4)	
H2	0.7494	0.9092	0.5047	0.049*	
C3	0.5949 (3)	0.78677 (18)	0.58188 (8)	0.0402 (4)	
H3	0.7026	0.7079	0.5747	0.048*	
C4	0.4029 (3)	0.78533 (16)	0.63518 (7)	0.0334 (3)	
C5	0.2448 (3)	0.90548 (17)	0.64295 (8)	0.0369 (4)	
C6	0.2657 (3)	1.02782 (17)	0.60204 (9)	0.0398 (4)	
H6	0.1562	1.1061	0.6088	0.048*	
C7	0.1208 (4)	0.75102 (19)	0.72418 (8)	0.0452 (4)	
H7	0.0293	0.7101	0.7619	0.054*	
C8	0.3173 (3)	0.68572 (17)	0.68946 (8)	0.0370 (4)	
C9	0.4300 (3)	0.54149 (18)	0.70437 (8)	0.0406 (4)	
H9A	0.3482	0.5023	0.7468	0.049*	
H9B	0.6166	0.5506	0.7136	0.049*	
C10	0.3882 (3)	0.44027 (15)	0.64264 (8)	0.0331 (3)	
N1	0.5982 (3)	0.38614 (19)	0.61193 (9)	0.0492 (4)	
O1	0.1637 (2)	0.41077 (13)	0.62188 (6)	0.0433 (3)	
O2	0.0692 (2)	0.88620 (13)	0.69762 (6)	0.0473 (3)	
O3	0.4914 (2)	1.14862 (11)	0.51112 (6)	0.0454 (3)	
HO3	0.5821	1.1314	0.4760	0.068*	

### Atomic displacement parameters (Å^2^)

**Table:**

	*U*^11^	*U*^22^	*U*^33^	*U*^12^	*U*^13^	*U*^23^
C1	0.0386 (8)	0.0371 (8)	0.0326 (7)	−0.0049 (6)	−0.0004 (6)	−0.0042 (6)
C2	0.0363 (9)	0.0486 (9)	0.0364 (7)	−0.0001 (7)	0.0078 (7)	−0.0003 (6)
C3	0.0371 (9)	0.0440 (8)	0.0396 (8)	0.0048 (7)	0.0066 (7)	−0.0023 (6)
C4	0.0306 (8)	0.0402 (8)	0.0294 (7)	−0.0038 (6)	0.0006 (6)	−0.0059 (6)
C5	0.0334 (8)	0.0444 (8)	0.0328 (7)	−0.0052 (7)	0.0050 (6)	−0.0093 (6)
C6	0.0381 (9)	0.0395 (8)	0.0417 (8)	0.0015 (7)	0.0042 (7)	−0.0081 (7)
C7	0.0499 (10)	0.0505 (9)	0.0353 (8)	−0.0090 (8)	0.0103 (7)	−0.0037 (7)
C8	0.0375 (9)	0.0449 (8)	0.0287 (7)	−0.0085 (7)	0.0000 (7)	−0.0056 (6)
C9	0.0411 (9)	0.0495 (9)	0.0312 (7)	−0.0058 (7)	−0.0054 (7)	0.0026 (6)
C10	0.0296 (8)	0.0377 (7)	0.0320 (7)	−0.0027 (6)	−0.0021 (6)	0.0070 (6)
N1	0.0320 (8)	0.0597 (9)	0.0559 (9)	−0.0004 (7)	0.0012 (7)	−0.0097 (8)
O1	0.0290 (6)	0.0567 (7)	0.0444 (6)	−0.0025 (5)	−0.0029 (5)	−0.0082 (5)
O2	0.0476 (7)	0.0508 (7)	0.0434 (6)	−0.0008 (6)	0.0187 (5)	−0.0079 (5)
O3	0.0537 (8)	0.0405 (6)	0.0419 (6)	−0.0003 (5)	0.0081 (5)	0.0008 (5)

### Geometric parameters (Å, º)

**Table:**

C1—O3	1.3717 (19)	C7—C8	1.341 (2)
C1—C6	1.383 (2)	C7—O2	1.385 (2)
C1—C2	1.401 (2)	C7—H7	0.9300
C2—C3	1.378 (2)	C8—C9	1.494 (2)
C2—H2	0.9300	C9—C10	1.511 (2)
C3—C4	1.398 (2)	C9—H9A	0.9700
C3—H3	0.9300	C9—H9B	0.9700
C4—C5	1.391 (2)	C10—O1	1.2390 (19)
C4—C8	1.447 (2)	C10—N1	1.317 (2)
C5—O2	1.372 (2)	N1—H2N	0.92 (3)
C5—C6	1.382 (2)	N1—H1N	0.92 (3)
C6—H6	0.9300	O3—HO3	0.8200
			
O3—C1—C6	116.76 (14)	C8—C7—H7	123.7
O3—C1—C2	121.52 (14)	O2—C7—H7	123.7
C6—C1—C2	121.69 (15)	C7—C8—C4	105.80 (15)
C3—C2—C1	120.98 (14)	C7—C8—C9	127.46 (16)
C3—C2—H2	119.5	C4—C8—C9	126.73 (15)
C1—C2—H2	119.5	C8—C9—C10	111.69 (12)
C2—C3—C4	118.87 (15)	C8—C9—H9A	109.3
C2—C3—H3	120.6	C10—C9—H9A	109.3
C4—C3—H3	120.6	C8—C9—H9B	109.3
C5—C4—C3	118.16 (14)	C10—C9—H9B	109.3
C5—C4—C8	105.84 (13)	H9A—C9—H9B	107.9
C3—C4—C8	136.00 (15)	O1—C10—N1	121.80 (15)
O2—C5—C6	124.99 (15)	O1—C10—C9	120.70 (14)
O2—C5—C4	110.44 (14)	N1—C10—C9	117.50 (15)
C6—C5—C4	124.56 (14)	C10—N1—H2N	121.9 (16)
C5—C6—C1	115.73 (15)	C10—N1—H1N	122.0 (17)
C5—C6—H6	122.1	H2N—N1—H1N	116 (2)
C1—C6—H6	122.1	C5—O2—C7	105.37 (13)
C8—C7—O2	112.56 (14)	C1—O3—HO3	109.5
			
O3—C1—C2—C3	177.50 (15)	O2—C7—C8—C4	−0.22 (19)
C6—C1—C2—C3	−0.7 (3)	O2—C7—C8—C9	178.51 (14)
C1—C2—C3—C4	−0.2 (2)	C5—C4—C8—C7	0.25 (17)
C2—C3—C4—C5	0.9 (2)	C3—C4—C8—C7	179.59 (18)
C2—C3—C4—C8	−178.39 (17)	C5—C4—C8—C9	−178.49 (14)
C3—C4—C5—O2	−179.68 (13)	C3—C4—C8—C9	0.8 (3)
C8—C4—C5—O2	−0.21 (17)	C7—C8—C9—C10	116.36 (19)
C3—C4—C5—C6	−0.8 (2)	C4—C8—C9—C10	−65.2 (2)
C8—C4—C5—C6	178.69 (15)	C8—C9—C10—O1	−59.9 (2)
O2—C5—C6—C1	178.69 (15)	C8—C9—C10—N1	119.67 (16)
C4—C5—C6—C1	−0.1 (2)	C6—C5—O2—C7	−178.81 (16)
O3—C1—C6—C5	−177.48 (14)	C4—C5—O2—C7	0.08 (17)
C2—C1—C6—C5	0.8 (2)	C8—C7—O2—C5	0.09 (18)

### Hydrogen-bond geometry (Å, º)

**Table:**

*D*—H···*A*	*D*—H	H···*A*	*D*···*A*	*D*—H···*A*
O3—H*O*3···O1^i^	0.82	1.92	2.7006 (16)	158
N1—H1*N*···O3^ii^	0.92 (3)	2.08 (3)	2.969 (2)	162 (2)
N1—H2*N*···O1^iii^	0.92 (3)	2.03 (3)	2.8958 (18)	156 (2)

Symmetry codes: (i) *x*+1/2, −*y*+3/2, −*z*+1; (ii) *x*, *y*−1, *z*; (iii) *x*+1, *y*, *z*.
